# Effectiveness, cost effectiveness, acceptability and implementation barriers/enablers of chronic kidney disease management programs for Indigenous people in Australia, New Zealand and Canada: a systematic review of mixed evidence

**DOI:** 10.1186/s12913-016-1363-0

**Published:** 2016-04-06

**Authors:** Rachel Reilly, Katharine Evans, Judith Gomersall, Gillian Gorham, Micah D. J. Peters, Steven Warren, Rebekah O’Shea, Alan Cass, Alex Brown

**Affiliations:** Wardliparingga Aboriginal Research Unit, South Australian Health and Medical Research Institute, Adelaide, Australia; Johanna Briggs Institute, Faculty of Health Sciences, University of Adelaide, Adelaide, Australia; Onemda VicHealth Koori Health Group, School of Population and Global Heath, the University of Melbourne, Carlton, Victoria Australia; Menzies School of Health Research, Darwin, Australia; Baker IDI Heart and Diabetes Institute, Alice Springs, Australia; School of Public Health, University of Adelaide, Adelaide, Australia

**Keywords:** Chronic kidney disease, Indigenous health, systematic review, chronic disease management

## Abstract

**Background:**

Indigenous peoples in Australia, New Zealand and Canada carry a greater burden of chronic kidney disease (CKD) than the general populations in each country, and this burden is predicted to increase. Given the human and economic cost of dialysis, understanding how to better manage CKD at earlier stages of disease progression is an important priority for practitioners and policy-makers. A systematic review of mixed evidence was undertaken to examine the evidence relating to the effectivness, cost-effectiveness and acceptability of chronic kidney disease management programs designed for Indigenous people, as well as barriers and enablers of implementation of such programs.

**Methods:**

Published and unpublished studies reporting quantitative and qualitative data on health sector-led management programs and models of care explicitly designed to manage, slow progression or otherwise improve the lives of Indigenous people with CKD published between 2000 and 2014 were considered for inclusion. Data on clinical effectiveness, ability to self-manage, quality of life, acceptability, cost and cost-benefit, barriers and enablers of implementation were of interest. Quantitative data was summarized in narrative and tabular form and qualitative data was synthesized using the Joanna Briggs Institute meta-aggregation approach.

**Results:**

Ten studies were included. Six studies provided evidence of clinical effectiveness of CKD programs designed for Indigenous people, two provided evidence of cost and cost-effectiveness of a CKD program, and two provided qualitative evidence of barriers and enablers of implementation of effective and/or acceptable CKD management programs. Common features of effective and acceptable programs were integration within existing services, nurse-led care, intensive follow-up, provision of culturally-appropriate education, governance structures supporting community ownership, robust clinical systems supporting communication and a central role for Indigenous Health Workers.

**Conclusions:**

Given the human cost of dialysis and the growing population of people living with CKD, there is an urgent need to draw lessons from the available evidence from this and other sources, including studies in the broader population, to better serve this population with programs that address the barriers to receiving high-quality care and improve quality of life.

## Background

Chronic Kidney Disease (CKD) occurs more frequently and in younger age groups amongst Aboriginal and Torres Strait Islander Australians than non-Indigenous Australians, with rates three to five times the national average in urban areas and up to 30 times the national average in remote areas [1]. Mortality is correspondingly high, with national data indicating CKD is a primary or associated cause of death in 16 % of Indigenous deaths [2]. A disproportionately high burden of CKD has also been found among First Nations people in Canada and Maori people in New Zealand, where there are similar persistent patterns of heath inequities between Indigenous and non-Indigenous people [3–5]. The incidence end-stage kidney disease (ESKD) has almost doubled between 1991 and 2008 and is projected to increase by 130 % from 2009 to 2020 [2, 6]. Dialysis is expensive, invasive, and leads to decreased quality of life, particularly for people living in rural and remote locations, who often have to leave their homes for extended periods and/or travel long distances to access treatment [7].

**Fig. 1:**
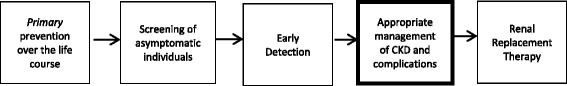
Focus of this review in relation to the prevention and management pathway for CKD

Reducing this burden will require cross-sector primary prevention strategies addressing risk factors across the life course, including increasing access to appropriate health care and early screening, [1, 8] as well as improved access to effective and acceptable treatment programs for those with CKD. This review is concerned with programs targeting Indigenous people with established CKD and as such has a deliberate focus on a narrow part of the treatment continuum [9] (Fig. 1). The review was initiated by renal staff working on the ‘front line’ in primary care settings in central Australia, who expressed an urgent need to find ways of stopping the rapid increase in ESKD, and in particular of understanding what may assist those with early-stage CKD to delay, or prevent, the need for dialysis. The goals of CKD management programs include reducing cardiovascular risk, identifying and managing complications, providing appropriate and timely interdisciplinary health-care, and supporting lifestyle modifications [9–12].

A recent systematic review of CKD programs in the United Kingdom, United States and Canada, found that care provided by a multidisciplinary team, compared to standard medical care, delayed the progression of CKD [13]. The four studies included in this review focused on education as the primary preventative strategy. A comprehensive preliminary search of relevant databases revealed that there is no existing systematic review examining evidence on the effectiveness, cost effectiveness, acceptability and/or implementation barriers/enablers of CKD management programs designed for Indigenous people living in Australia or elsewhere.

The present review sought to address the following questions:

1. What is the effectiveness of CKD programs designed for Indigenous people in relation to outcomes, including, though not limited to: clinical indicators of CKD management such as blood pressure control; the delayed progression of kidney disease/time to dialysis; and quality of life?
2. What are the costs and costs relative to benefits of CKD programs designed for Indigenous people from the perspectives of individual patients and their families, the primary health services that deliver them, tertiary health services and society as a whole?
3. What do patient and provider experiences of CKD programs designed for Indigenous people reveal about the acceptability of programs, as well as barriers and enablers of implementation?

## Methods

A protocol for the review was published in the Joanna Briggs Institute (JBI) Database of Systematic Reviews and Implementation Reports [14]. This project was developed in accordance with the National Health and Medical Research Council’s Values and Ethics: Guidelines for ethical research in Aboriginal and Torres Strait Islander Research [15], and with the South Australian Aboriginal Health Research Accord [16].

### Search and study selection

The search strategy sought published and unpublished studies in English, published between 2000 and 2014. Earlier studies were considered less relevant due to advances in technology and data collection. Following the JBI and Cochrane guidelines [17, 18], a four-step search strategy was designed with a view to accessing the most relevant published literature, and also took into account the large amount of Indigenous health research evidence contained in grey literature [19] (Table 1).

**Table 1:** Four-step search strategy

Step	Search strategy
1	Limited search of PubMed and CINAHL, analysis of text words in titles and abstracts and of index terms used to describe the articles
2	Search using all identified keywords and index terms across all included databases: PubMed, EBSCO CINAHL, Embase, ATSIHealth via Informit online, Web of Science, Psychinfo, Social Science Citation Index, APAIS Health databases, Australian Indigenous Health InfoNet and Primary Health Care Research and Information Service (PHCRIS), Mednar, Trove, Google Grey, OCLC WorldCat Dissertations and Theses, Canada Theses Portal and other sources: websites of relevant organizations in each country including Kidney Health Australia, Kidney Health New Zealand and The Kidney Foundation of Canada, Australian Institute of Torres Strait Islander Studies, NativeWeb and World Health Organization^a^
3	Search of reference lists of all identified reports and articles for additional studies
4	Search of all relevant published systematic reviews and consultation with experts

^a^Searches for each database available from the authors

The PubMed search strategy is shown in Table 2.

**Table 2:** Pubmed search terms

Search	Query
#1 Population of Interest	(Australia[mh] OR Australia*[tw] OR.au[ad] OR Australia*[ad] OR Northern Territory[tw] OR Northern Territory[ad] OR Tasmania*[tw] OR Tasmania*[ad] OR New South Wales[tw] OR New South Wales[ad] OR Victoria*[tw] OR Victoria*[ad] OR Queensland[tw] OR Queensland[ad] OR Canada[mh] OR Canad*[tw] OR.ca[ad] OR Canad*[ad] OR Alberta[tw] OR Alberta[ad] OR British Columbia[tw] OR British Columbia[ad] OR Manitoba[tw] OR Manitoba[ad] OR New Brunswick[tw] OR New Brunswick[ad] OR Newfoundland and Labrador[tw] OR Newfoundland and Labrador[ad] OR Northwest Territories[tw] OR Northwest Territories[ad] OR Nova Scotia[tw] OR Nova Scotia[ad] OR Nunavut[tw] OR Nunavut[ad] OR Ontario[tw] OR Ontario[ad] OR Prince Edward Island[tw] OR Prince Edward Island[ad] OR Quebec[tw] OR Quebec[ad] OR Saskatchewan[tw] OR Saskatchewan[ad] OR Yukon Territory[tw] OR Yukon Territory[ad] OR New Zealand[mh] OR New Zealand[tw] OR.nz[ad] OR New Zealand[ad] OR Aotearoa[tw]) AND (Oceanic ancestry group[mh] OR American Native continental ancestry group[mh] OR Maori[tw] OR Aborig*[tw] OR indigenous[tw] OR (Torres Strait[tw] AND Islander*[tw]) OR Inuit*[tw] OR eskimo*[tw] OR native[tw] OR First Nation*[tw])
#2 Disease	kidney diseases[mh] OR chronic disease[mh] OR chronic kidney[tw] OR chronic renal[tw] OR predialysis[tw] OR pre dialysis[tw] OR albumin creatinine ratio[tw] OR estimated glomerular filtration rate[tw] OR diabetic nephropath*[tw]
#3 Intervention or Setting	disease management[mh] OR health services, indigenous[mh] OR rural health[mh] OR rural population[mh] OR rural health services[mh] OR preventive health services[mh] OR community networks[mh] OR delivery of health care[mh] OR health planning[mh] OR case management[tw] OR intervention[tw] OR management[tw] OR service*[tw] OR model*[tw] OR program*[tw] OR multidisciplinary[tw] OR co ordination[tw] OR coordination[tw] OR integrated[tw] OR transdisciplinary[tw] OR participatory[tw] OR community[tw] OR care[tw] OR prevent*[tw] OR health education[tw] OR health promotion[tw] OR exercise[tw] OR rural[tw] OR outreach[tw] OR remote[tw] OR focus group*[tw] OR ambulatory[tw] OR general practice[tw] OR clinic[tw] OR primary[tw] OR outpatient[tw] OR telemedicine[tw]
#4	#1 AND #2 AND #3
Limits:	Publication date from 01/01/2000–2014; English language.

The search results were imported into the EndNote (Thomson Reuters) citation manager and pooled into a single library. After removing duplicates, titles and abstracts were screened against the inclusion criteria by two reviewers (RR and KE) working independently. Those articles clearly not meeting the inclusion criteria were excluded. The full texts of the remaining articles were examined and those meeting the inclusion criteria were assessed for methodological quality.

### Assessment of quality of included studies

Two reviewers (RR and KE) assessed methodological quality using relevant Joanna Briggs Institute (JBI) standardized critical appraisal instruments [17]. Quantitative papers examining CKD program effectiveness were assessed using the tools contained in the ‘JBI Meta-Analysis of Statistics Assessment and Review Instrument’ (JBI-MAStARI). JBI-MAStARI has separate tools for appraising different study designs. In the absence of a specific tool tailored for appraisal of uncontrolled before and after studies, these were appraised using the ‘descriptive/case series’ appraisal tool. Studies of costs and cost effectiveness were assessed using the tools contained in the ‘JBI Analysis of Cost, Technology and Utilization Assessment and Review Instrument’ (JBI-ACTUARI). Qualitative papers were assessed using the tool in the ‘JBI Qualitative Assessment and Review Instrument’ (JBI-QARI).

Any disagreements that arose between the reviewers were resolved through discussion or with a third reviewer (JG). Studies were classified as good quality if more than 80 % of appraisal characteristics were endorsed, moderate quality if 50–80 % of characteristics were endorsed and poor quality if less than 50 % were endorsed. Those rated as poor quality were excluded.

### Data extraction

Quantitative, economic and qualitative data were extracted from papers included in the review by three reviewers (RR, KE and JG) who worked independently using the standardized data extraction tools from JBI-MAStARI, JBI-ACTUARI and JBI-QARI [17]. Details about study characteristics (e.g., interventions, populations, settings and study methods) were extracted, as well as findings for the outcomes/phenomena of interest relevant to the review. Authors were contacted where necessary to clarify reported data or access information not reported.

### Data synthesis

The findings from included quantitative studies were synthesized using narrative and tables. The findings of qualitative studies were synthesized using the JBI meta-aggregation synthesis tool in JBI-QARI. This involved the aggregation of findings to generate a set of representative statements and categorizing the findings on the basis of similarity in meaning. These categories were then subjected to a meta-synthesis to produce a single, comprehensive set of synthesized findings [17, 20]. The heterogeneity of studies in terms of interventions, populations, reported data and study designs precluded both meta-analysis and second level aggregated synthesis. As such, the results for each question are presented separately below.

## Results

### Search and study selection

**Fig. 2:**
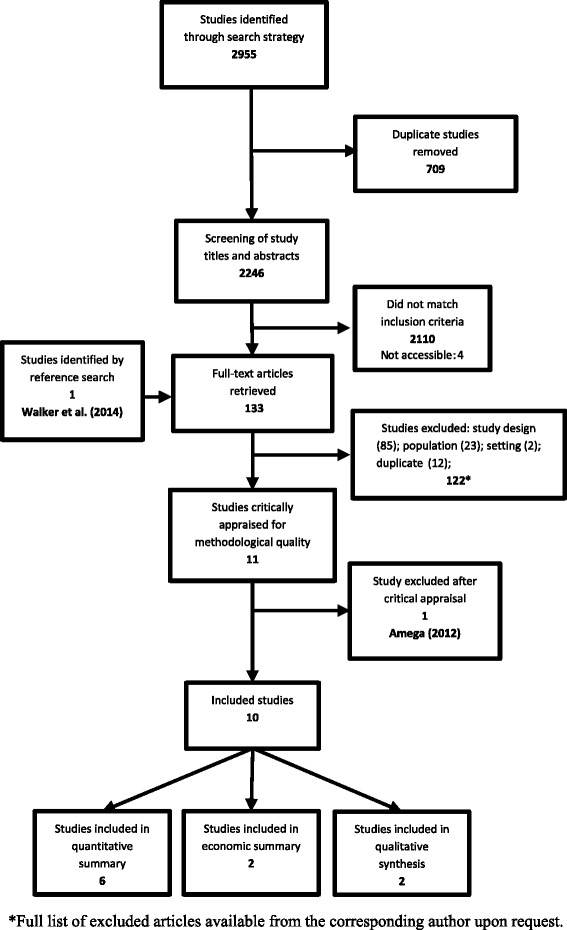
PRISMA Diagram: Search and Study Selection

As shown in Fig. 2, the search returned 2246 unique citations that were screened by title and abstract against the review inclusion criteria. The full texts of 136 papers were then reviewed independently by the primary and secondary reviewer (RR and KE), and reference lists checked for additional relevant articles. Checking the reference lists yielded one additional article that was included for full-text examination. Of these 137 articles, 85 were excluded on the basis of study design, 23 on the basis of population of interest, 2 were conducted in inpatient settings and 12 were duplicates (eg. where there were multiple publications from the same study). Four articles were not accessible after extensive efforts to access electronically and contacting the authors. A list of the studies excluded at full text examination with reasons for exclusion is provided as an additional file. Of the ten included studies, six provided quantitative evidence addressing the question of intervention effectiveness, two on costs (1) and cost effectiveness (1), two provided qualitative evidence on barriers/enablers on CKD program implementation and one study provided quantitative evidence on CKD program acceptability.

### Methodological quality of included studies

The majority of articles were rated as moderate quality, two were good quality and one study of poor quality was excluded [21]. Common weaknesses included a lack of randomisation and insufficient follow-up period for quantitative studies, a lack of clarity around the credibility of values assigned to costs and outcomes for economic studies, and the absence of a statement about the cultural or theoretical position of the researcher, or addressing their influence on the research for qualitative studies. The critical appraisal tools are published in full elsewhere [17] (Table 3).

**Table 3:** Critical appraisal of studies meeting the inclusion criteria

	RCT	Hotu et al. (2010) [22]	Comparable Cohort	Kondalsamy-Chennakesavan (2003) [26]	Descriptive/Case Series	Tan et al. (2014) [23]	Walker et al. (2014) [25]	Walker et al. (2013) [24]	Amega (2012) [21]	Shephard et al. (2006) [27]	Qualitative	Walker et al. (2012) [34]	Tchan et al. (2012) [33]	Economic	Gador-Whyte et al. (2014) [31]	Baker et al. (2005) [30]
Q1.	Was the assignment to treatment groups truly random?	Y	Is the sample representative of patients in the population as a whole?	Y	Was the study based on a random or pseudo-random sample?	N	N	N	N	N	There is congruity between the stated philosophical perspective and the research methodology?	Y	U	Is there a well-defined question?	Y	Y
Q2.	Were participants blinded to treatment?	N	Are the patients at a similar point in the course of their condition?	N	Were the criteria for inclusion in the sample clearly defined?	Y	Y	Y	Y	Y	There is congruity between the research methodology and the research question or objectives?	Y	Y	Is there a comprehensive description of alternatives?	NA	NA
Q3.	Was allocation to treatment groups concealed from the allocator?	Y	Has bias been minimized in relation to selection of cases and controls?	U	Were confounding factors identified and strategies to deal with them stated?	N	Y	Y	N	N	There is congruity between the research methodology and the methods used to collect data?	Y	Y	Are all important and relevant costs and outcomes for each alternative identified?	Y	U
Q4.	Were the outcomes of people who withdrew described and included in the analysis	Y	Are confounding factors identified and strategies to deal with them stated?	Y	Were outcomes assessed using objective criteria?	Y	Y	Y	Y	Y	There is congruity between the research methodology and the representation and analysis of data?	Y	Y	Has clinical effectiveness been established?	NA	Y
Q5	Were those assessing outcomes blind to the treatment allocation?	N	Are outcomes assessed using objective criteria?	Y	If comparisons are being made, were there sufficient descriptions of the groups?	NA	NA	NA	N	NA	There is congruence between the research methodology and the interpretation of results?	Y	Y	Are costs and outcomes measured accurately?	Y	Y
Q6.	Were the control and treatment groups comparable at entry?	Y	Was follow-up carried out over a sufficient time period?	Y	Was follow-up carried out over a sufficient time period?	N	N	N	U	N	There is a statement locating the researcher culturally or theoretically	N	N	Are costs and outcomes valued credibly?	Y	U
Q7.	Were groups treated identically other than for the named interventions?	Y	Were the outcomes of people who withdrew described and included in the analysis?	Y	Were the outcomes of people who withdrew included in the analysis?	N	Y	Y	N	Y	The influence of the researcher on the research, and vice-versa, is addressed	N	N	Are costs and outcomes adjusted for differential timing?	U	Y
Q8.	Were outcomes measured in the same way for all groups?	Y	Were outcomes measured in a reliable way?	U	Were outcomes measured in a reliable way?	Y	Y	Y	U	N	Participants and their voices are adequately represented	Y	Y	Is there an incremental analysis of costs and consequences?	N	Y
Q9.	Were outcomes measured in a reliable way?	Y	Was appropriate statistical analysis used?	Y	Was appropriate statistical analysis used?	Y	Y	Y	NA	Y	The research is ethical according to current criteria or evidence of ethical approval by an appropriate body	Y	Y	Are sensitivity analyses conducted to investigate uncertainty in estimates of cost or consequences?	N	Y
Q10.	Was appropriate statistical analysis used?	Y									Conclusions drawn in the research report appear to flow from the analysis or interpretation of the data	Y	Y	Do study results include all issues of concern to users?	Y	U
Q11.														Are the results generalizable to the setting of interest in the review?	U	U
	Quality Rating^a^	8/10 Good		6/9 Moderate		4/8 Moderate	6/8 Moderate	6/8 Moderate	2/8 Poor	4/8 Moderate		8/10 Good	7/10 Moderate		5/9 Moderate	6/10 Moderate

*Y* yes, *N no*, *U* unclear. ^a^Good: at least 80 %; Moderate: 50-80 %; Poor: less than 50 %

## Findings on the effectiveness of CKD programs

### Study characteristics

As outlined in Table 4, of the six studies providing quantitative evidence on program effectiveness, four were conducted in New Zealand [22–25] and two in Australia [26, 27]. Four of these were uncontrolled prospective cohort designs carried out over one [24, 25] or 2 years [23, 27]. Of a number of possible publications reporting effectiveness of the Menzies Renal Treatment Program (MRTP), the thesis by Kondalsamy-Chennakesavan [26] was selected because it was the most relevant and comprehensive, including two comparisons: 1) before and after the MRTP was handed over to the Tiwi Health Board (THB); and 2) outcomes from the MRTP versus the THB-run Continuing Care Trial (CCT). Australian participants were younger, on average, than the New Zealand participants (weighted averages 44.1 years and 57.8 years respectively). The 437 participants overall were split evenly between men and women (49.9 % men).

**Table 4:** Characteristics of studies addressing question 1

Study	Objective	Study design	Setting	Intervention and comparator	Comparator	Participants	Outcomes measured
Tan et al. (2014) [23]	To determine the effectiveness of a PHC-based, nurse-led CKD program with Tongan staff can improve medication adherence and clinical outcomes	2-year prospective uncontrolled cohort study, conducted 2011 – 2013	NZ urban area, PHC service in Auckland with Tongan-speaking staff	Nurse-led with input from GP and diabetologist when necessary. Focus on prescribing antihypertensives and improving adherence. BP measured 2–6 weekly. Some outreach and lifestyle, dietary and self-care education.	No comparator.	43 Pasifika patients with type 2 diabetes, CKD (mostly stages 2 and 3) and hypertension. Mean age 53 years, 77 % male. 39 available for follow-up at ≥17 mths.	BP, no. antihypertensives, eGFR, ACR, HbA1c
Walker et al. (2013, 2014) [24, 25]	To test feasibility and effectiveness of a specialist renal nurse-led self-management intervention to slow progression of CKD.	1 year prospective uncontrolled cohort study, conducted 2011–2012.	NZ, rural area; two PHC practices in Hawke’s Bay.	Specialist nurse-led partnership with primary care clinicians. Focus on coaching to improve self-management. Individual educational and clinical care plans developed followed by 12 weeks of fortnightly self-management sessions, with monitoring to 12 months. Some outreach and free care, medications and transport.	No comparator.	52 patients (37 NZ Māori, 10 Cook Island Māori/Samoan and 5 NZ European) with type 2 diabetes, CKD	BP, no. antihypertensives, eGFR, ACR, HbA1c, self-management.
Hotu et al. (2010) [22]	To determine whether a nurse-led community-based CKD program involving a Māori or Pasifika health care assistant (HCA) (‘community care’; CC) is more clinically effective than ‘usual care’ (UC).	1 year RCT, conducted 2004–2006.	NZ, urban area; hospital clinics and PHC services in Auckland.	Nurse-led with focus on prescribing antihypertensives and improving adherence. Monthly outreach by HCA to monitor BP, promote adherence and provide free transport. Lifestyle, dietary and self-care education. Received routine care as necessary.	Lifestyle, dietary and self-care education. Usual care by GP and renal clinic.	65 Māori and Pasifika patients with type 2 diabetes, CKD (mostly stage 3) and hypertension (CC: *n* = 33; UC: *n* = 32). Mean age: CC: 63; UC: 60 years; % male: CC: 55 %; UC: 53 %. 58 available for follow-up at 12 months (CC: *n* = 30; UC: *n* = 28).	BP, no. antihypertensives, adherence, eGFR, ACR, HbA1c.
Shephard et al. (2006) [27]	To determine the clinical effectiveness (and acceptability- see below) of the Umoona Kidney Project, a PHC-based partnership between the local Aboriginal community controlled health service (ACCHS) and visiting specialists from Adelaide.	2 year prospective uncontrolled cohort study, conducted 1998–2000.	Australia, remote area; ACCHS in Coober Pedy.	Specialist-run with focus on prescribing antihypertensives, delivering ACR point of care tests (POCT) and ascertaining acceptability of project. Regular visits by nephrologists and 6-monthly monitoring of clinical parameters. Lifestyle and dietary education provided. Some outreach.	No comparator.	35 Aboriginal patients with hypertension and with or at risk of CKD (20 had albuminuria). Mean age 49 years, 54 % male. Patients followed for a mean of 15 months with none lost to follow-up.	BP, no. antihypertensives, adherence, eGFR, ACR, program acceptability.
Kondalsamy-Chennakesavan (2003) [26]	1) To determine whether improvements in BP and metabolic control were sustained following the handover of the visiting specialist-run MRTP to the local THB. 2) To compare the effectiveness of the pre-handover MRTP to the concurrently run THB-managed CCT.	2.5 and 5.5 year retrospective uncontrolled cohort study, comparing cohorts: 1) 66 month MRTP cohort (*n* = 101) comparing pre-handover (1995–1999) and post-handover (2000–2001). 2) 30 month MRTP (*n* = 149) and CCT (*n* = 89) cohorts comparing pre-handover MRTP to CCT (1997–2000).	Australia, remote area; ACCHS on the Tiwi Islands, 80 km north of Darwin.	The MRTP was a specialist-run project that ran alongside the local health care facilities. The focus was prescribing antihypertensives. Lifestyle and dietary education delivered and individual treatment plans developed. Systematic recalls and active follow-up to monitor BP.	CCT patients assigned a chronic disease care plan and were managed in routine PHC setting. No specific resources for renal patients, opportunistic follow-up, less systematic medical oversight.	238 Aboriginal patients with hypertension and/or CKD (mostly stages 1 and 2). Mean age: MRTP: 44; CCT: 42 years; % male: MRTP: 45 %; CCT: 44 %.	BP, HbA1c.

#### Findings

Studies reported clinical indicators of CKD management including blood pressure control, the use of anti-hypertensive medication, albumin-creatinine ratio (ACR), glycemic index (HbA1c) and glomerular filtration rate. No studies reported data on hard end-points such as dialysis or death. There were also no data reported on quality of life or other psychosocial variables, such as depression or stress.

Relevant clinical outcomes from all studies are shown in Table 5. All intervention groups showed significant reductions in systolic blood pressure from baseline or in relation to comparator groups. All groups who reported on ACR reported reductions, although this was non-significant in Shephard [27] This study was limited by very small sample size. Where it was reported, glycated hemoglobin was lower at follow-up for most programs, except for Kondalsamy-Chennakesavan [26], where results were mixed following the handover of the program to the Tiwi Health Board when it was incorporate into routine primary health care. Findings relating to estimated glomerular filtration rate (eGFR) are also mixed. eGFR is an important indicator of CKD function but is complicated as a measure of program effect because it can decrease in the short term with use of antihypertensive medications. For many people with CKD, GFR reduces steadily over time, while for others it may follow a non-linear trajectory [28].

**Table 5:** Findings relating to question 1

	Tan et al. (2014) [23]		Walker et al. (2013, 2014) [24, 25]		Hotu et al. (2010) [22]		Shephard et al. (2006) [27]		Kondalsamy-Chennakesavan (2003) [26] #1		Kondalsamy-Chennakesavan (2003) [26] #2	
Outcome measure (n)	Baseline (*n* = 43)	17mths (*n* = 39)	Baseline (*n* = 52)	12mths (*n* = 36)	Baseline CC(*n* = 33) UC(*n* = 32)	12mths (*n* = 30) (*n* = 28)	Baseline	15mths	Baseline MRTP (*n* = 149) CCT (*n* = 89)	30mths (*n* = 149) (*n* = 89)	Pre-(*n* = 101)	Post-(*n* = 101)
Systolic Blood Pressure mmHg(SD)	137 (17)	126 (16)*	153 (15)	131 (11)*	161 (20) 161 (20)	140 (19) 149 (23)**	151 (18)	137 (18)*	132 (22) 126 (20)	123 (16) 128 (16)**	124 (14)	129 (15)
Median ACR mg/mmol(IQR) ^#^g/day(IQR)	126 (65–194)	51 (20–97)	34.9 (14.2–150.9) Mean:^c^ 134.5 (286.5)	Median not reported Mean: 44.7 (76)*	3.3 (1.5–3.2) 1.6 (0.9–4.0)	2 (0.5–3.8) 3.3 (1.5–5.3)**	5.7 (1.2–15.2)	4.3 (1.3–16.7)	NA	NA	NA	NA
eGFR	68 (50–81)	63.1 (42–73)*	63.1 (20.2)	60.8 (18.2)	39 (14) 36 (15)	41 (18) 33 (17)	110	118*	NA	NA	NA	NA
HbA1c %(SD)	9.6 (24)	8.6 (20)*	9.1 (14)^b^	8.0 (9)^b^*	8.3 (9)^a^ 8.5 (11)^a^	8.0 (10)^a^ 7.9 (9)^a^	NA	NA	NA	NA	NA	NA

*p < 0.05 from baseline to follow-up

**p < 0.05 program vs. comparator at follow-up in Hotu et al. (2010) [22]

#Hotu et al. (2013) measured 24h urinary protein

^a^SE converted to SD (SD = N√SE)

^b^Mmol/mol converted to %

^c^Means provided by author. Change per unit per month −0.34 (−0.55, −0.12), *p* < 0.05

Indicators of health knowledge and behavior were not widely measured. Hotu et al. [22] measured medication adherence by self-report questionnaire, and found that 80 % took their medication ‘most of the time’ in the intervention arm, compared to 71 % in usual care. In Shephard et al. [27], tablet counts indicated that 72 % of participants took their medication at least 80 % of the time. Walker et al. [24, 25] measured ‘self-management’ from the perspectives of patients and clinicians using 13 questions of the Partners in Health instrument [29] and reported improvements in 12 of 13 domains.

## Findings on the cost and cost effectiveness of CKD programs

### Study characteristics

Two studies provided evidence relating to costs or cost-effectiveness of CKD programs from the perspective of service providers. Baker et al. [30] measured the cost-effectiveness of the Menzies Renal Treatment Program (MRTP), which was also included in the quantitative review [26], and Gador-Whyte et al. [31] compared the estimated the costs of delivering best-practice care, as defined by Central Australian Rural Practitioners’ Association (CARPA) guidelines [32], with actual expenditure for patients with Type 2 diabetes and/or CKD in an Aboriginal Community Controlled Health Service in remote Central Australia.

#### Findings

When comparing the MRTP to usual care, Baker et al. [30] found that the risk of starting dialysis in the treatment group relative to historical controls over a 4.7 year period was reduced by 57 % (*p* = 0.03), as shown in Table 6. Moreover, that over the 4.7 years, 36.8 person years of dialysis were avoided by implementing the MRTP. The reduced number of dialysis starts generated net savings of $4.2 million (in 1997–1998 AUD). Sensitivity analysis indicated that these findings were robust to changes in costing assumptions (Table 7).

**Table 6:** Characteristics of studies addressing question 2

Study	Objective	Study design	Setting	Intervention and comparator	Comparator	Participants	Outcomes measured
Gador-Whyte et al. (2014) [31] *Cost of Best practice care*	To estimate, from a remote ACCHS perspective, the cost of completing best practice chronic care tasks for patients with type 2 diabetes and/or CKD.	Partial economic evaluation/costing study.	Australia, remote area; ACCHS in unnamed Central Australian Aboriginal community.	Best practice care for patients with diabetes and/or CKD.	Usual care delivery for patients with diabetes and/or CKD in that particular ACCHS setting	*Patients*: 205 Aboriginal patients: 74 had diabetes, 86 had CKD and 45 had both.	Costs: annual costs (total and per patient) of managing CKD and diabetes in 2009–2010 and projected annual costs using optimal PHC management; difference in these actual and projected costs.
						*ACCHS staff*: 4 AHWs, 3 nurses, 1 GP, 1 educator, 1 exercise physiologist.	
						Conducted 2010–2011.	
Baker et al. (2005) [30] * Menzies Renal Treatment Program*	To assess, from a government health service perspective, if the MRTP reduced the costs of treating ESKD through improved clinical outcomes.	Economic evaluation.	Australia, remote area; ACCHS on Tiwi Islands, 80 km north of Darwin.	Program to modify kidney and cardiovascular disease. Antihypertensives and health education offered.	Usual Care	*Intervention group*: 258 Aboriginal patients with hypertension and/or CKD.	Health outcomes: Dialysis starts and dialysis person-years avoided.
						*Comparator group*: 229 Aboriginal patients in a historical control group (1992–1995).	Costs: MRTP delivery costs; ESKD treatment costs; total cost.
						Conducted 1995–2000.	Net cost of the program/savings compared to usual care.
							Measured at 3 and 4.7 years.

**Table 7:** Comparison of the effects and costs of the MRTP and control at 4.7 years (Baker et al.) [26]

	MRTP	Control	Difference
Number of client years	897.8	897.8	
Program delivery cost (incremental)	$987,926	$0	$987,926
*Endpoint: ESKD treatment*			
ESKD treatment years incurred	27.7	64.5	−36.8
ESKD treatment costs incurred	$3,120,350	$7,265,796	–$4,145,446
Total cost (program and ESKD costs)	$4,108,276	$7,265,796	–$3,157,521
*Endpoint: dialysis start*			
Relative risk for treatment versus control	0.43 (0.19–0.96), *p* = 0.012		
*Reduction* in risk of starting dialysis in the treatment versus control	57 %, *p* = 0.03		
Number of dialysis starts	11	26	−15
Lifetime ESKF treatment costs incurred	$3,853,332	$9,107,875	–$5,254,543
Total cost (program and lifetime ESKD costs)	$4,841,258	$9,107,875	–$4,266,618

Gador-Whyte et al. [31] reported a total funding gap of $198,728 per annum or $1733 per patient between the projected cost of best practice care and actual expenditure in 2009–2010. No sensitivity analysis was conducted, therefore it is unclear whether funding gaps of similar magnitudes have applied, and continue to exist, in other ACCHS and community settings with different staffing and cost structures. The study also identified workforce shortages, low health literacy and a high acute care workload as factors that may prevent delivery of best practice care (Table 8).

**Table 8:** Costs of usual and best practice care for patients in an ACCHS setting (Gador-Whyte et al.) [27]

	Estimated 2009–10 costs ($)		Projected best practice costs ($)		Difference ($)	
Costs for diabetes and CKD care in a remote ACCHS	*Annual*	*Per patient (mean)*	*Annual*	*Per patient (mean)*	*Annual*	*Per patient (mean)*
	446,585	6123	645,313	7856	−198,728	−1733

## Findings on acceptability and barriers/enablers of implementation

### Study characteristics

The two qualitative studies included in the review, from Australia and New Zealand, described experiences of CKD programs/models of care to Indigenous people from the perspective of providers. Characteristics of included qualitative studies are shown in Table 9. Tchan et al. [33] evaluated the Outback Vascular Health Service (OVHS), described as a chronic disease outreach program. The study documents service provider experiences of providing care to Aboriginal people with CKD, which reveal barriers and enablers of implementing acceptable and effective CKD care to Aboriginal people. Walker et al. [34] is a descriptive, exploratory study describing pre-dialysis nurses’ experiences of delivering care to CKD patients on outpatient clinics. It offers evidence relating to barriers and enablers to providing effective CKD care to Maori and Pasifika patients.

**Table 9:** Characteristics of studies addressing question 3

Study	Study design	Setting	Participants	Study objectives
Tchan et al. (2012) [30]	Mixed methods study. Qualitative component used a descriptive, exploratory approach. Semi-structured interviews and inductive analysis. Conducted 2009–2012.	Australia, remote area; ACCHS in Broken Hill and surrounding towns.	20 service providers comprising 4 medical specialists, 6 managers, 2 Aboriginal health workers (AHWs), 5 GPs, 3 local Aboriginal employees.	To understand provider views on the implementation of the Outback Vascular Health Service (OVHS), a chronic disease outreach program that operated regularly within the Maari Ma ACCHS
Walker et al. (2012) [34]	Descriptive, exploratory approach. In-depth semi-structured interviews and thematic analysis guided by Thomas’ (2006) general inductive approach.	NZ, variety of areas; pre-dialysis clinics primarily on the North Island.	11 pre-dialysis nurses working with large case-loads of clients approaching ESKD, including a significant proportion of Māori and Pasifika patients.	To understand perceptions of pre-dialysis specialist nurses on factors influencing their delivery of effective pre-dialysis care.
Shephard et al. (2006) [26]	7-item Cross-sectional survey measured on a 5-point scale and administered by either AHWs, the nurse in charge, community leaders or a medical student	Australia, remote area; ACCHS in Coober Pedy	50 community members including 27 participants in the Umoona kidney program	To determine the acceptability of the Umoona Kidney Project

Shephard et al. [27] present the results of a brief survey of program acceptability developed for the Umoona Kidney Program. This 7-item questionnaire was administered to 50 Aboriginal community members, including the participants in the program. Items such as *‘Are you happy with the way the kidney team treats you?’* were measured on a 5-point scale from ‘very much yes’ to ‘very much no.’

#### Findings

**Fig. 3:**
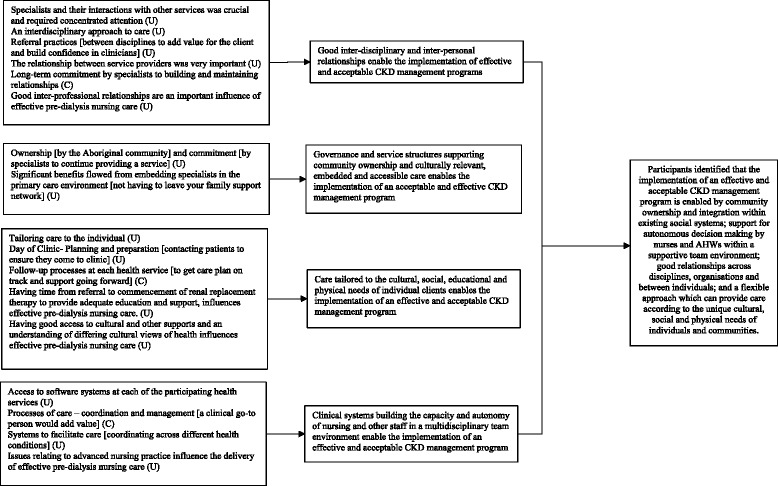
Synthesis of qualitative findings addressing question 3

Twenty-nine findings on barriers or enablers of CKD program implementation were identified and extracted from the two qualitative studies. Twelve unsupported findings (those without supporting data) in Tchan et al. [33] were excluded. The 17 remaining findings, summarized in Fig. 3, were grouped into four categories. These categories were synthesized into one overall finding. There was no qualitative evidence from the perspectives of patients.

The survey of acceptability reported by Shepard et al. [27] found very positive attitudes amongst community members towards the Umoona Kidney Project. However, the small sample and bias inherent in the phrasing of questions and mode of administration make this questionnaire difficult to interpret with confidence.

## Discussion

The purpose of this review was to examine evidence on the effectiveness, cost effectiveness and acceptability CKD management programs for Indigenous people, as well as barriers and enablers of implementation. Given the broad scope of these questions, which were directed at providing guidance for the design and implementation of future CKD programs, the inclusion criteria were broad and inclusive in regards to the types of programs and outcomes considered. In light of this, the small number of included studies, in particular addressing questions 2 and 3, was surprising. An examination of the studies excluded from this review indicates that there is a lot written but not a lot of it is intervention research. While the heterogeneity, small sample sizes and moderate quality of the included studies limit generalizability, the findings do indicate that targeted CKD programs are effective in improving clinical outcomes for Indigenous people with CKD, such as maintaining blood pressure within target ranges and reducing HbA1c and albuminuria. Alongside data on the effectiveness of programs, the inclusion of evidence on cost, cost-effectiveness, acceptability, and barriers and enablers of implementation was considered important for informing the future program development, given the unique economic, social and cultural contexts in which CKD programs for Indigenous peoples are implemented. The small quantity of research addressing these questions suggests that little is currently known about the how CKD management programs for Indigenous peoples are experienced, in particular from the perspectives of clients, their families or communities; and also indicates that economic evaluation is not routinely included in CKD program evaluation. Nonetheless the body of evidence included in the review provides a useful indication of the particular features that should be considered in the design of CKD management programs for Indigenous people.

All programs were multifaceted. While it is not possible to draw conclusions about the particular components of programs that may be causally related to improved outcomes, we identified characteristics common to many of the programs. Future qualitative and quantitative research could explore these questions more fully. Common components of effective programs were: the integration or coordination with primary care; nurse-led or Indigenous Health Worker-led care; intensive follow-up including home-visits; the provision of anti-hypertensive medication following a step-wise protocol; and addressing barriers to adherence such as cost and lack of transport. Education also emerged as a key component of effective programs, but it had to be delivered in ways that accounted for literacy and culture. These program features were consistent with the findings addressing the question of acceptability, and barriers and enablers of implementation. There is also overlap between these findings and evidence in non-Indigenous populations, which indicates that nurse-led and/or multidisciplinary [13, 35], protocol-driven [36] care embedded in primary health care and including patient education tends to lead to better outcomes [13, 35, 36].

To address the question of cost and cost effectiveness (Q2), we sought to identify studies of CKD management programs that considered cost and cost-effectiveness in their evaluations. Such studies involve weighing up factors and conditions specific to a particular time and context. As such, our ability to draw generalizable conclusions from the two included studies is limited. For example, the effectiveness data in Baker et al. [30] should be considered in light of the medical advances that have occurred in routine practice since the data was collected between 1995 and 1998 (although the study was published in 2005). Similarly, Gador-Whyte et al. [31] conducted their study in 2010–2011, and since that time relevant changes to the funding structures of Aboriginal Community Controlled Health Organizations have occurred, such as the introduction of the Practice Incentive Program (PIP) Indigenous Health Incentive, which provides eligible Indigenous health services with a payment for each patient who is registered for chronic disease care, and an additional payment for those who receive a target level of care in a calendar year [37, 38].

Nonetheless, the studies provide some insights relating to the resourcing of CKD programs. Gador-Whyte et al. [31] provide a valuable outline of what constitutes best-practice care in a remote Aboriginal Health Service. The funding short-fall between projected (best-practice) and actual expenditure appeared across clinical staff, administrative staff and other operating costs. It was noted that an acute work-load, health literacy, under-staffing and high staff turnover were barriers to the provision of best-practice care, reflecting broader issues such as the challenge of recruiting and retaining staff in remote communities, rather than a funding shortfall per se. Baker et al. [30] suggest that from a government perspective, the Menzies Renal Treatment Program was a ‘good buy’ for health, as it generated improvement in health outcomes and reduced suffering for patients, as well as monetary savings by reducing dialysis start numbers. The positive impact on quality of life is the stronger and, arguably, sufficient argument for investing in primary and secondary prevention programs. Furthermore, effective management of CKD is likely to have additional benefits for the prevention of other acute and chronic health problems (such as cardiovascular disease), leading to other social and economic benefits that should be taken into account.

Regarding the question of acceptability, and barriers and enablers of implementation (Q3), the two studies included in this review reveal important enablers to implementing CKD management programs to Indigenous people such as governance structures that support community ownership and culturally relevant care; flexible care that can meet the needs of people in their particular context; and robust clinical systems that support communication, staff autonomy and capacity building. In particular, the important role of nurses and Indigenous Health Workers was again highlighted in both studies. These program features are in line with Gibson et al. [39] who found that community engagement, coordination of care, embedding culturally safe care, for example by employing Indigenous people, and respecting patients’ perspectives enabled the implementation of chronic disease care. Excluding IHWs from decision-making and poorly performing electronic support systems were barriers to implementation.

## Conclusions

Overall, the findings of this review point to the benefit of CKD care that caters to people in their social and cultural environments. That is, care that is embedded within existing healthcare services that people already use. Health services utilising intensive outreach aim to remove barriers to adhering to medical regimens and attending appointments, as well as enabling education and assisting with goal setting in an appropriate location for individuals and families. The role of nurses and/or Indigenous health workers was emphasised in delivering effective outreach and education, and the role of primary health care services was emphasised, in line with recent policy recommendations [39–41].

There is no doubt that more rigorous evaluations of programs over longer time-frames would assist a better understanding the longer-term effectiveness and sustainability of CKD programs, and to understand the mechanisms by which programs lead to change. Researchers should also be encouraged to adhere to best practice by outlining program theories, to enable the assessment of program fidelity, and a better understanding of why and how a program effect occurs. However, given the human cost of dialysis and the growing population of people living with CKD, there is a critical need to draw lessons from the available evidence, including this and other sources in Australia and internationally, to better serve Indigenous people with programs that address the barriers to receiving high-quality care and improve quality of life.

## Abbreviations

ACCHS: Aboriginal community controlled health service
ACR: Albumin-creatinine ratio
CKD: chronic kidney disease
eGFR: estimated glomerular filtration rate
ESKD: end-stage kidney disease
HbA1c: Glycated hemoglobin
JBI: Joanna Briggs Institute
JBI-ACTUARI: JBI analysis of cost, technology and utilization assessment and review instrument
JBI-MAStARI: JBI meta-analysis of statistics assessment and review instrument
JBI-QARI: JBI qualitative assessment and review instrument
MRTP: Menzies renal treatment program
SBP: systolic blood pressure
THB: tiwi health board
